# Rapid Prototyping of Soft Lithography Masters for Microfluidic Devices Using Dry Film Photoresist in a Non-Cleanroom Setting

**DOI:** 10.3390/mi10030192

**Published:** 2019-03-15

**Authors:** Prithviraj Mukherjee, Federico Nebuloni, Hua Gao, Jian Zhou, Ian Papautsky

**Affiliations:** 1 Department of Bioengineering, University of Illinois at Chicago, Chicago, IL 60607, USA; pmukhe5@uic.edu (P.M.); hgao22@uic.edu (H.G.); jzhou88@uic.edu (J.Z.); 2 Department of Electronics, Informatics and Bioengineering, Politecnico di Milano, 20133 Milan, Italy; nebuloni.federico@gmail.com

**Keywords:** dry photoresist, soft lithography, microfluidics

## Abstract

Fabrication of microfluidic devices by soft lithography is by far the most popular approach due to simplicity and low cost. In this approach PDMS (polydimethylsiloxane) is cast on a photoresist master to generate replicas that are then sealed against glass slides using oxygen plasma. In this work, we demonstrated fabrication of soft photolithography masters using lamination of ADEX dry film as an alternative to the now classic SU-8 resist masters formed by spin coating. Advantages of using ADEX dry film include the easily-achievable uniform thickness without edge bead; simplicity of the process with significant time savings due to non-sticky nature of the film; and fewer health concerns due to less toxic developing solution and antimony-free composition. As we demonstrate, the process can be performed in a low-cost improvised fabrication room in ambient light, in place of a conventional yellow-light cleanroom environment. We believe this approach holds the promise of delivering state-of-the-art microfluidic techniques to the broad field of biomedical and pharmaceutical research.

## 1. Introduction

Explosive growth of microfluidics in the past two decades has led to development of a number of fabrication approaches. Microfluidic chips are now commonly fabricated in glass by wet etching [1,2] or in polymers by milling [3,4,5], injection molding [6], hot embossing [7], laser ablation [8], and most recently 3D printing [9,10,11]. A significant drawback of these techniques is that most require expensive instrumentation and/or a cleanroom setting. Thus, soft lithography for microfluidic device fabrication [12] remains the most popular fabrication method due to simplicity and ease of sealing. The approach involves casting of PDMS (polydimethylsiloxane) on a photoresist mold formed by photolithography and can be accomplished without extensive instrumentation. 

The most common technology enabling soft lithography is to pattern molds using SU-8 photoresist [12,13,14,15]. SU-8 is a negative-tone i-line resist available in a wide range of viscosities, permitting a wide range of thicknesses and geometries [16]. However, one downside is that it is difficult to obtain good flatness of the resist, potentially leading to non-uniform channel structure. This non-uniformity arises from the edge bead—a buildup of resist at the wafer edge as resist flows outward form wafer center under the influence of centrifugal force during spin coating. The common solution to this is edge bead removal using organic solvent. The disadvantage, however, is that in a poorly controlled process there can be considerable splashing and misting of the solvent leading to resist coating defects. 

Dry film resists (DFRs) were developed for the printed circuit board fabrication and offer many advantages over liquid photoresists, such as SU-8. First and foremost, DFRs offer excellent flatness and uniformity, no formation of edge bead, and no necessity for liquid resist handling. Other potential advantages include shorter processing time, lower cost, and nearly-vertical sidewalls. Further, the setup costs for DFR processing are significantly lower than that for liquid photoresists. Indeed, a number of investigators have begun reporting use of DFRs for MEMS (microelectromechanical systems) and microfluidic device fabrication, including electroplating molds, microchannels, digital microfluidic devices, or hybrid chips [17,18,19]. 

In this work, we demonstrate fabrication of soft lithography masters using an antimony-free ADEX dry film photoresist (DJ MicroLaminates, Inc., Sudbury, MA, USA). The ADEX films are well suited for creating microfluidic channel masters 5–75 µm, with channels then formed using the conventional PDMS soft lithography process. The ADEX films offer excellent surface uniformity, good adhesion to substrate, processing simplicity, and prototyping in a non-cleanroom setting. 

## 2. Experimental Methods

### 2.1. Photolithography Fabrication Process

**Figure 1:**
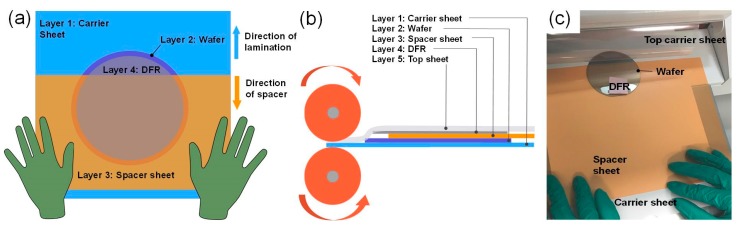
Dry film photoresist lamination procedure. (**a**) Top view schematic of the film-wafer lamination assembly. The top carrier sheet of the lamination pouch is not shown; (**b**) side view schematic of the film-wafer lamination assembly. The top carrier sheet (layer 5) of the lamination pouch is shown here; (**c**) photograph of the assembly prior to lamination: Carrier sheet (white), spacer sheet (orange) and DFR (pink) are illustrated. The spacer sheet (layer 3) is held as the rollers pull the assembly into the laminator. The spacer sheet ensures gradual lamination and avoids formation of air bubbles. The top and bottom carrier layers (layers 1 and 5) protect the DFR from sticking to the rollers and any dust particles.

Following dehydration, each wafer was laminated with ADEX film. First, wafer was placed on the bottom carrier sheet of the lamination pouch (PM Company, Fairfield, OH, USA, via Amazon), with a 0.68 mm PET (polyethylene terephthalate) spacer sheet 1–2 cm from the top edge of the wafer. The “X” side of the protective layer of the dry film was peeled off exposing the adhesive side of the film. The ADEX film was then placed with the adhesive side on to the exposed wafer top, pressing down gently with one finger to secure film to the wafer. The film was placed such that the adhesive side does not adhere to the spacer sheet. The film-wafer sandwich lamination assembly is shown in Figure 1, illustrating the order of the aforementioned films. Once secure, the film-wafer sandwich was fed into a hot roller laminator (SKY 325R6, Sky DSB, Seoul, Korea via Amazon), at a feed rate of ~0.3 m/min, with the roller temperature set to 65 °C. Note that ADEX films must be handled by hand only and not with tweezers, as the metal surface may scratch or break the delicate films. Direction of motion of the carrier and spacer sheets is shown in Figure 1a. As the rollers pull the film assembly into the laminator, the spacer sheet (layer 3) is held by the operator to prevent it from being pulled in. The spacer sheet ensures gradual lamination on the wafer from top to bottom and minimizes formation of any bubbles or folds. 

After lamination, the wafer was placed on a hot plate for soft baking at 65 °C for 5–10 min. The exact bake time is dependent on film thickness and is given in Table 1. The carrier and spacer sheets were wiped with a lint free wipe wetted in acetone and blow dried with nitrogen, to remove any dust particles that may accumulate in between steps. 

The laminated wafers were exposed through a mask containing channel patterns using 169–637.2 mJ/cm^2^ collimated UV light (Optical Associates Inc., San Jose, CA, USA). The exact exposure time is dependent on film thickness and is given in Table 1. A glass filter to cut off wavelengths above 365 nm was used. Following exposure, the wafer was placed on a hot plate for post-exposure bake (PEB) at 95 °C for 5–10 min. The exact bake time is dependent on film thickness and is given in Table 1. After PEB, the wafer was developed in cyclohexanone (Acros Organics, Pittsburg, PA, USA) in a glass Petri dish agitated mildly on an orbital shaker (Mini Orbital Shaker, VWR, Radnor, PA, USA). The developed wafers were then washed with IPA and DI water, followed by blow drying. Respective development times are given in Table 1. The wafers were then hard baked on a hot plate at 150 °C for 90 min to improve adhesion. 

### 2.2. Channel Fabrication by Soft-Lithography

Multiple masks were used in this work to fabricate microchannels and test patterns. Masks were designed using AutoCAD (Autodesk, Inc., San Rafael, CA, USA) and were fabricated in chrome-coated glass and Mylar film (Front Range PhotoMask Co., Lake Havasu City, AZ, USA). One photomask contained test features that included circular arrays of 100 μm and 50 μm diameter pillars, placed 45 μm and 20 μm apart, as well as arrays of 100 μm and 50 μm wide rectangles placed 10, 15, 20, and 25 μm apart. Other photomasks contained microfluidic channels for inertial separations or blood fractionation we used in our previous studies. These channels ranged in width from 10 to 500 μm and varied in shape from straight to spiral to serpentine. Channels and test features were fabricated by standard soft-lithography process, using PDMS (polydimethylsiloxane, Sylgard 184, Dow Corning Midland, MI, USA). Briefly, PDMS was mixed with a curing agent at a ratio of 10:1, degassed, and then poured onto the masters. The wafers with PDMS were degassed again before curing at 75 °C for 2 h. PDMS replicas peeled from the masters and either cut for measurement of cross-sectional dimensions or sealed to a glass microscope slide with oxygen plasma for microfluidic testing. 

### 2.3. Imaging and Analysis

Completed wafers were imaged using a digital microscope with a zoom lens (Sciencescope International, Chino, CA, USA) to inspect wafer quality and features. PDMS replicas and cross-section samples were imaged using an inverted microscope (Olympus IX-83, Olympus America Inc., Lombard, IL, USA) fitted with a high-resolution sCMOS camera (Zyla 5.5, Andor, Concord, MA, USA). The images were later analyzed and measured using CellSense imaging software (Olympus America Inc., Lombard, IL, USA). 

## 3. Results and Discussion

### 3.1. Microfluidic Fabrication in a Non-Cleanroom Setting

A fabrication room was improvised to replace a conventional yellow-light cleanroom environment. ADEX does not appear to have much sensitivity to ambient light, and in our experience no light filtration is necessary to process it. This is due to the low spectral intensity of the conventional compact fluorescent bulbs near 365 nm (UVA), while the new LED bulbs generally emit light above 400 nm. Thus, any lab space with ambient light could be used, although spaces with windows and sunlight should be avoided. A laminar flow hood with HEPA filtration can be used on top of a bench to provide additional particulate control and improve the process, which is vulnerable to dust during the baking steps when wafers are resting on top of a hotplate. Alternatively, an oven can be used for baking steps and to shield wafers from ambient dust.

The ADEX films were laminated using a low-cost photo-pouch heated-roller laminator. The SKY 325R6 model (Sky DSB, Seoul, Korea via Amazon) used in this work has a 12.8-inch opening, which is more than sufficient to laminate a range of substrates, including 4” and 6” wafers. Alternative models of the laminator with other opening widths are also commercially available. The key features of the laminator are the precise temperature control and the ability to digitally input the desired temperature. A feed rate of 0.3 m/min was used in this work. A slow feed rate may cause melting of the film whereas a faster feed rate may result in improper adhesion of the film to the wafer and may also cause air bubbles being trapped under the film. 

For UV exposure, a low-cost collimated UV light source (Optical Associates Inc., San Jose, CA, USA) replaced a mask aligner. The 500 W light source provided ~20 mW/cm^2^ beam intensity at 365 nm, with approx. 98% beam uniformity and approx. 2° beam divergence. The system is well suited for single-layer photoresist fabrication, as light collimation permits vertical sidewalls when working with thick, >25 µm photoresist layers, and feedback circuitry allows for constant intensity of the exposed area. A programmable shutter timer is also useful for precise control of the exposure time. An even lower-cost solution (<$500) would be to replace the collimated light source with a simple UV lamp [18,19]. However, such light sources do not offer precise timing control and can lead to non-vertical sidewalls in thick photoresist films due to a lack of light collimation. Ultimately, regardless which UV light option is used, a mask is placed directly on top of the dry film laminated onto silicon wafer. Both chrome plates and low-cost Mylar films were used as masks. In the latter case, a clean glass plate (e.g., an old mask plate with chrome stripped) can be used to keep the Mylar mask perfectly flat and in contact with resist during exposure. 

The ADEX-laminated substrates were developed in ambient light inside a conventional fume hood, due to cyclohexanone emitting odor reminiscent to that of acetone. The final step in microfluidic fabrication is to seal PDMS microchannels. While this process is not specific to the dry film process, its discussion is relevant in the context of fabrication in a non-cleanroom setting. While the conventional process involves 20% oxygen plasma treatment in a reactive ion etching system (RIE) [20], which are widely available in most cleanrooms, a low-cost solution is to use a corona discharge wand to treat both PDMS and glass surfaces in ambient air [3]. While this approach works well for low-pressure microfluidic applications (e.g., PDMS channels for cell culture), the formed bond is not sufficiently strong for higher pressure applications (e.g., cell separations [21,22] or flow cytometry [23]). Herein, we used a cost-effective plasma system (PlasmaEtch Inc., Carson City, NV, USA) that offers oxygen treatment under vacuum and is sufficiently compact to be placed on a benchtop. 

### 3.2. Photoresist Performance and Characterization

Multiple microfluidic devices and test structures were fabricated in PDMS using ADEX resist masters to validate the fabrication process. The smallest features that were reproduced reliably were 10 µm wide channels in 10 and 20 µm thick films (aspect ratio AR = 1–2). While some dry resists have been reported to have difficulties reproducing smaller features, possibly due to light scattering associated with not using a collimated UV light source [18], we encountered no difficulties in replicating gap structures of 10 µm in width in these films. The process parameters reported in Table 1 yielded the best results.

**Figure 2:**
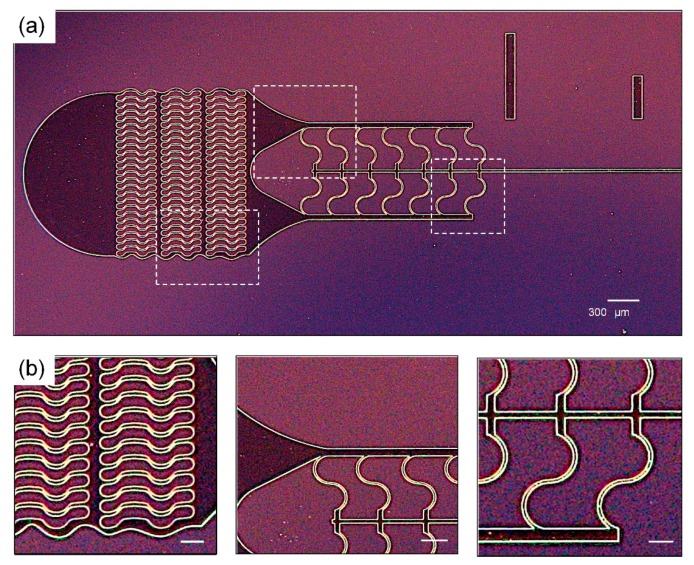
Representative images of a completed ADEX master using optimized process parameters. (**a**) Image of the channel inlets filters, with 30 μm spacing between each filter pillar. The scale bar is 300 μm; and (**b**) close-up images of different channel sections. The scale bar is 50 μm.

The resist yielded excellent definition of microfluidic structures that span a range of sizes, such as input filters we [24] and others [25] use in microfluidic devices to prevent channel clogging when working with particulate flows. Figure 2 shows a top view of such a filter, implemented in a 50 µm thick film and composed of wavy pillars 100 µm wide and spaced 30 µm apart, followed by a segment of S-shaped structures 30 µm wide, prior to a 30 µm wide microchannel. The film exhibited good adhesion and these critical channel features did not deform or get stripped off the wafer post development or washing. 

Surface modification of the resist to prevent adhesion during PDMS soft lithography was not necessary due to hydrophobic surface. A siliconizing reagent, such as Sigmacote (Sigma-Aldrich, Milwaukee, WI, USA) is typically used in SU-8 fabrication [26] to prevent adhesion of PDMS to freshly fabricated masters. These reagents are generally based on polysiloxane in solvent that readily form a covalent, microscopically thin film on surface of SU-8, making it strongly hydrophobic. This step is critical if ashing (brief O_2_ plasma treatment) is used to clean-up post-development resist residue. The ADEX films, however, did not exhibit difficulty in release of PDMS, likely due to its hydrophobic nature post development (contact angle −94° ± 2). We performed an estimated 100 castings over several weeks and did not observe any significant damage or distortion of features on the masters with the minimum feature size of 10 µm.

**Figure 3:**
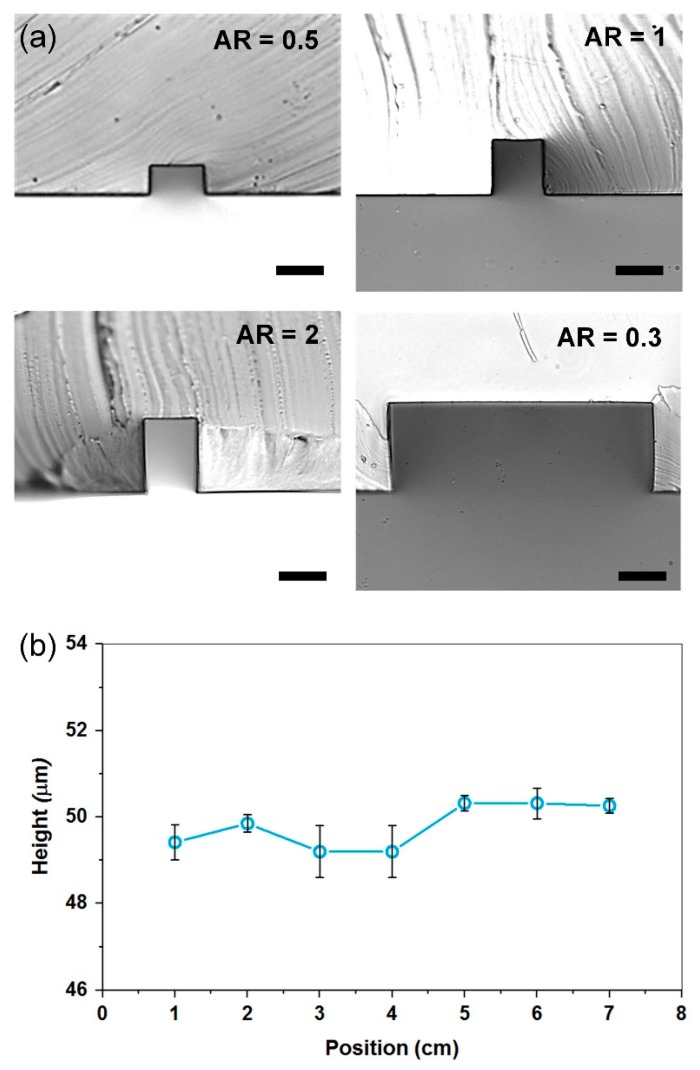
PDMS replicas of ADEX masters. (**a**) Channel cross-sections of various aspect ratios. Scale bar is 20 µm; and (**b**) the average channel height measured along the entire length of the wafer, with coefficient of variation of ~1.1% (*n* = 35).

Masters with different heights were fabricated to assess performance range of the ADEX film. Figure 3a shows cross-sections of different aspect ratio, illustrating precise rectangular shape and perpendicular walls. The images were obtained by forming a PDMS replica of the laminated film and then slicing through it in order to image its cross-section. Flatness of the ADEX film, and the resulting microchannel downstream uniformity, was measured by slicing PDMS channel replica in 1 cm increments. The data for a 7 cm long channel, spanning the entire 3” wafer (Figure 3b) show a uniform 49.9 µm thickness. Variability across the entire channel was approx. 1.6 µm. The SU-8 fabricated devices have been reported to have variability of approximately 5 µm across wafer surface [18]. 

**Figure 4:**
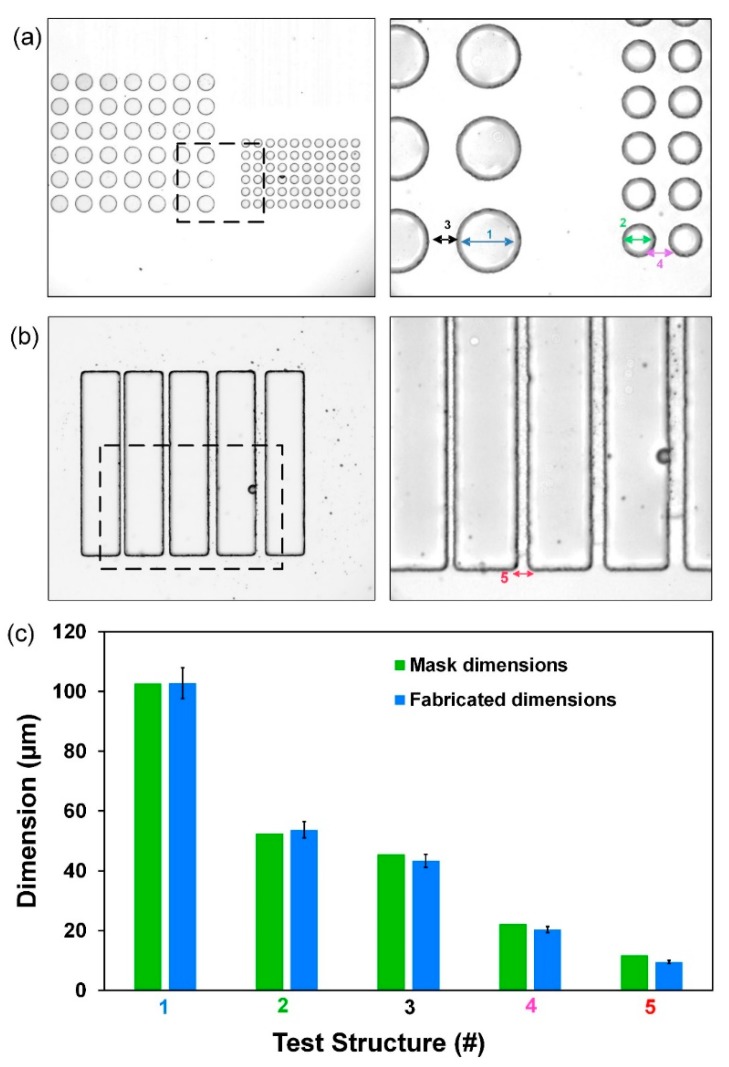
PDMS replicas of test structures in ADEX resist to test resolution, accuracy and adhesion. Dimensions are those commonly used in microfluidic devices for biomedical applications. (**a**) Circular pillar arrays of diameter 100 µm (Test Structure 1) and 50 µm (Test Structure 2), separated by 45 µm (Test Structure 3) and 20 µm (Test Structure 4) gaps; (**b**) rectangular pillar array separated by 10, 15 (Test Structure 5), 20 and 25 µm gaps; and (**c**) comparison of dimensions in the high-resolution mask vs. fabricated features in dry film (*n* = 25).

To better assess resolution and quantify the minimum features size, we designed test structures using circular posts (Figure 4a) of different diameters (100 and 50 µm), spaced at different gap distances (50 and 25 µm). We also used an array of 100 µm wide rectangular pillars (Figure 4b) spaced at different gaps width (10, 15, 20, and 25 µm). Measurements from PDMS replicas show that the completed wafers have dimensions within ±2 μm of the dimensions of the mask (Figure 4c). 

**Figure 5:**
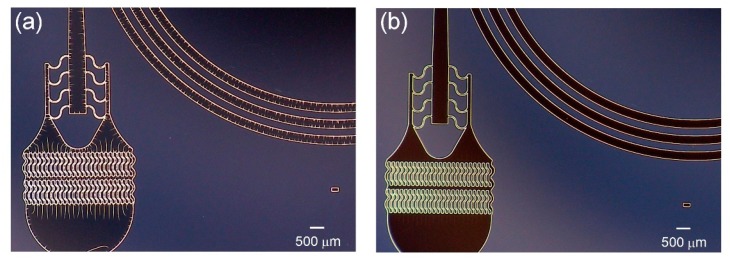
Images of an ADEX master before and after annealing. (**a**) Sub-micron cracks appear upon development in cyclohexanone in some films. (**b**) However, these cracks can be removed by annealing at 150 °C for 90 min.

Stress in the photoresist film manifested as cracks on the surface or irregular lines near edges. This can be clearly observed in Figure 5a. However, unlike earlier work with Ordyl SY300/550 dry film resists [18], no loss of substrate adhesion was observed. The stress appears to depend on the PEB settings, as well as development in cyclohexanone due to a fast drop in temperature from 95 °C to room temperature. In our case, however, cracks can be completely removed with a hard bake step at 150 °C on a hot plate for 90 min after development. This is shown in Figure 5b. This crack recovery might be due to a softening of the resist features, with the heat that allows cracks to be resorbed without changing their shape, in addition to strengthening the resist adhesion with the substrate and the robustness of the film against aggressive chemistry and handling.

**Figure 6:**
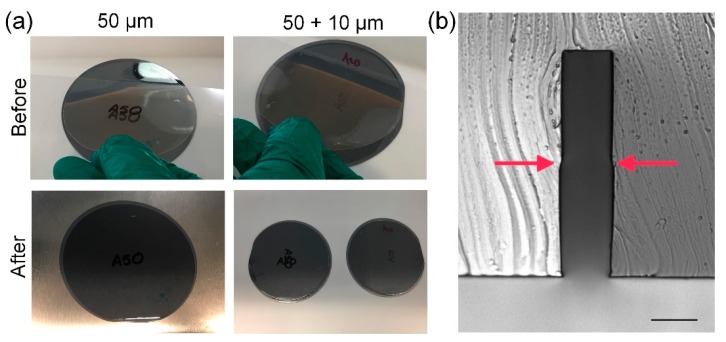
Images before and after lamination of double layered films. (**a**) First layer lamination using 50 μm ADEX film on a clean silicon wafer. After completion, top protective film is peeled off and a second layer of 10 μm thick ADEX film is placed directly on top; and (**b**) cross-sectional cut of PDMS cast on the double-laminated ADEX master. Arrows point to the joint between the layers of two 50 µm films. The scale bar is 25 µm.

While ADEX resist is available in 5 to 75 µm thick films, other film thicknesses maybe achieved by combining films. For example, we double-laminated two 50 μm films to achieve a 100 μm thickness, as shown in Figure 6. Adhesion between consecutive ADEX films is strong, resulting in a single structure in the end, without any adverse discontinuities. The same roller speed (0.3 m/min) was used in both laminating steps. No air-bubbles between two layers were observed. It should be noted, however, that the double lamination process is not cost effective, as the cost of a 100 µm thick film is not significantly higher than that of a 50 µm film. However, there is a degree of convenience in being able to form films of any desirable thickness for one-off prototyping or when the specific film is not readily available. 

### 3.3. Comparison with Conventional Photoresist

A key advantage of the dry film lamination process is that it eliminates the need for spin-coating, thus providing a cleaner and less time-consuming approach to achieving film thickness with excellent uniformity. Although the popularity of liquid photoresist is unquestionable, it is a relatively complex technique that requires experience to successfully handle it, mainly due to a few parameters that can impact final results. Table 2 summarizes the advantages and disadvantages of dry film over SU-8 for fabrication. For SU-8, a number of optimization or tweaking runs are generally necessary to achieve desired height and vertical sidewalls. In particular, lower height features can only be fabricated with low viscosity SU-8 (e.g., SU-8 TF 6000), which are even more affected by moisture and temperature variations, resulting in a very low adhesion behavior on silicon [27]. Spin coating is highly sensitive to the spinning speed applied for coating, as even a minimum alteration of the speed can lead to a sensible variation in the height of the coating, which may have drastic effects for further application of the master. Although tuning capability is an undisputed strength of this technique, it can produce height variations depending on the accuracy of the spin coater used and if speed is not correctly estimated from MicroChem Corp. datasheets, and some trials are necessary to set the process. In addition to the precision required in each step of the fabrication protocol, SU-8 is sensitive to the environmental conditions of the fabrication room, such as humidity and temperature. Dry films however provide more flexibility and hassle-free conditions. From the setup standpoint there is no need for a snorkel/vented exhaust as the cost of modifying duct work can often exceed the cost of the spin coater. Conversely, lamination process of dry films is a cleaner approach than having to deal with the post process cleaning of splattered SU-8 in the spin coater. 

During exposure of SU-8 coated wafers, a small volume of glycerol is often added to the surface to reduce potential light refraction due to the non-uniform SU-8 surface caused by edge bead and to protect the mask from sticking. If this care is not taken, the SU-8 sticking to the mask may polymerize, making it difficult to separate post-exposure, and requiring aggressive cleaning of the mask. In the case of DFR, this limitation is overcome as the film does not stick to the mask even after UV exposure. Both chrome and Mylar masks can be used in direct contact with the film, reducing the gap between mask and wafer. Ultimately, this can result in significant time savings. 

It is also worth mentioning that ADEX film is developed in cyclohexanone. Although this step must be performed inside a fume hood due to odor similar to that of acetone, cyclohexanone is relatively less toxic than propylene glycol monomethyl ether acetate (PGMEA)-based developers used with SU-8 and other DFRs (Table 2). Cyclohexanone is commonly used as a precursor to nylon and can be purchased from any laboratory vendor at relatively low cost, which is an advantage over other photoresists that use custom developers available only from photoresist manufacturers. Additionally, SU-8 also contains antimony [28,29,30], long-term exposure to which may have detrimental health effects [31], while ADEX is antimony-free [32]. 

Although the photolithographic lamination technique with ADEX dry films offers a simpler processing alternative to the classic SU-8 photolithography, it is not without some limitations. First, the substrate options are limited. While the ADEX film has excellent adhesion to silicon, it does not however adhere well to glass. PMMA, polycarbonate or polystyrene, which are popular polymer fabrication materials, also cannot be used as they are not chemically compatible with the cyclohexanone developer. Nevertheless, silicon is the commonly used substrate material for photoresist masters is soft lithography and, thus, from the microfluidic fabrication standpoint this is not a significant limitation.

Secondly, while it is theoretically possible to overlap multiple ADEX films before UV exposure, as we demonstrate, lamination of DFR on a substrate already containing previously formed features or significant texture is not possible. Conversely, this can be done with SU-8 to form multi-layer masters and structures [33], as its liquid form permits conformal coating. Another limitation is related to the tuning capability, if the spin-coated SU-8 allows the user to finely modify the thickness of the resist layer by simply changing the spinning speed, ADEX dry films are fixed to the purchased batch. It can be challenging to change the thickness of the laminated layer to a height other than the ones provided by the vendor other than summing multiple dry films (Table 2). To our knowledge, the thinnest films available are 5 μm, so this approach is not be suitable for applications that require thinner films. 

## 4. Conclusions

In this work, we demonstrated fabrication of soft photolithography masters using lamination of ADEX dry film photoresist as an alternative to the now classic SU-8 resist masters formed by spin coating. Advantages of using ADEX are threefold: (1) it is a clean process that makes uniform thickness easily achievable; (2) it is a simple process with significant time savings due to non-sticky nature of the dry film; and (3) it affords fewer health concerns due to a less toxic developing solution and its antimony-free composition. The process can be performed in a low-cost improvised fabrication room in ambient light, in place of a conventional yellow-light cleanroom environment. The ADEX film is comparable to other dry films currently on the market, such as SY300 (Ordyl), MX-5000 or WLP-1000 (DuPont), and SUEX (DJ MicroLaminates), in terms of the processing approach. However, most of these films are similar to SU-8 in that they use developing solutions based on PGMEA, which is a highly flammable and toxic compound. Ultimately, ADEX films offer excellent surface uniformity, good adhesion to substrate, nearly vertical sidewalls, processing simplicity, and non-cleanroom setting prototyping-characteristics that make ADEX well-suited for fabrication of microfluidic channel masters.

## Figures and Tables

**Table 1:** Process parameters for ADEX films.

Thickness (μm)	Soft Bake at 65 °C (min)	PEB at 95 °C (min)	Exposure Energy (mJ/cm^2^)	Exposure Time (s)	Development Time (min)
10	2 to 3	3 to 5	169	14	4.5
20	2 to 3	3 to 5	223	21	5
25	3 to 5	3 to 5	255	24	6
50	5 to 10	5 to 10	350	33	20
75	5 to 10	5 to 10	425	40	25
100	10	10–12	650	60	35

**Table 2:** Comparison of ADEX and SU-8 photoresists for microfluidic device fabrication.

Key Features for Master Fabrication	ADEX Process	SU-8 Process
Processing in a non-cleanroom environment	Yes	No
Good adhesion to silicon wafer	Yes	Yes
No need for descumming plasma treatment	Yes	No *
Uniform thickness without edge bead	Yes	No
Flexibility of height	Low	High
Uniformity over entire wafer	High	Low
Process cleanliness	Yes	No **
Need to siliconize wafer post development **	No	Yes ***
Low-cost setup	Yes	No
Level of expertise needed for rapid prototyping	Low	High
Developer and film toxicity	Low	High

* Plasma treatment for SU-8 is generally needed for descumming the surface, although not always required; ** Spin-coating is messy and requires periodic cleaning of the coater bowl; *** It is generally necessary for SU-8 structures to be siliconized following descumming oxygen plasma treatment, although may not be required if plasma treatment is not used.
